# The predictive role of the neutrophil–lymphocyte ratio in the prognosis of adult patients with stroke

**DOI:** 10.1186/s41016-020-00201-5

**Published:** 2020-07-01

**Authors:** Jinzhao Wan, Xiaoxiong Wang, Yunbo Zhen, Xin Chen, Penglei Yao, Wenwu Liu, Enzhou Lu, Yiming Du, Huailei Liu, Shiguang Zhao

**Affiliations:** 1 Department of Neurosurgery, The First Affiliated Hospital of Harbin Medical University, No. 23 Youzheng Street, Nangang District, Harbin, 150001 Heilongjiang Province People’s Republic of China; 2 Institute of Brain Science, Harbin Medical University, No. 23 Youzheng Street, Nangang District, Harbin, 150001 Heilongjiang Province People’s Republic of China; 3 Institute of Neuroscience, Sino-Russian Medical Research Center, Harbin Medical University, No. 23 Youzheng Street, Nangang District, Harbin, 150001 Heilongjiang Province People’s Republic of China

**Keywords:** Prognosis of stroke, adult patients with stroke, Neutrophil–lymphocyte ratio, Acute stroke, Predictive role

## Abstract

Our study aimed to determine the effect of the neutrophil–lymphocyte ratio on the prognosis of adult patients with acute stroke. We searched the Web of Science, PubMed, Embase, Cochrane Library, and China National Knowledge Infrastructure databases and selected all of the potentially eligible studies. From the included studies, we extracted characteristics such as the stroke type and acquisition time until routine blood collection and the odds ratios across studies. The 95% confidence intervals and odds ratios were pooled to calculate the effect size for the neutrophil–lymphocyte ratio in acute stroke patients. We defined poor function outcomes according to the modified Rankin Scale ≥ 3 or Glasgow Outcome Scale< 3.Thirteen studies with 4443 patients were included in our analysis, including 7 ischemic and 6 hemorrhagic stroke studies. The pooled odds ratios for poor functional outcome at 3 months with a higher neutrophil–lymphocyte ratio in acute ischemic and hemorrhagic patients were 1.689 (95% CI = 1.184–2.409, *p* < 0.001) and 1.125 (95% CI = 1.022–1.239, *p* < 0.001), respectively, and the overall pooled odds ratio for poor functional outcome following stroke was 1.257 (95% CI = 1.146–1.379, *p* < 0.001). At the same time, the overall combined odds ratio for death at 3 months was 1.632 (95% CI = 1.155–2.306, *p* < 0.001).The neutrophil–lymphocyte ratio, an easily calculated marker, plays a predictive role in the short-term outcomes of adult patients (mean age ≥ 50 years) following acute ischemic and hemorrhagic stroke.

## Background

Stroke is a medical event in which the brain loses its function because of abnormal blood supply [1]. In general, we divide stroke into two categories: ischemic and hemorrhagic strokes, based on the distribution of brain blood. It has been reported that ischemic stroke accounts for approximately 85% of strokes [2], and it is characterized by a disruption in cerebral blood flow [3], while hemorrhagic stroke is characterized by bleeding within the intracranial space [4]. In addition to the characteristics of the haematoma, such as volume and location [5, 6], other factors, such as blood pressure variability [7] and cholesterol and ferritin levels [8, 9], and brain imaging parameters [10] have been reported to be related to the prognosis of hemorrhagic stroke patients. In ischemic stroke, infarct volume and location influence the severity of prognosis. Recently, several studies have reported that the neutrophil–lymphocyte ratio (NLR) may be a good marker of prognosis acute stroke (AS) patients at 3 months [11–14].

In the epidemiological statistics of the Global Burden of Disease, stroke is the second leading cause of mortality and physical disability in the world [2], and the overall burden of stroke has dramatically increased [15], especially in developing countries with low and middle incomes [16]. The neutrophil–lymphocyte ratio, a conventional marker, may be regarded as a prognostic factor in the short-term outcome of adult patients who suffer from acute stroke [11–14, 17]. Therefore, we systematically performed a meta-analysis to assess the prognostic role of the NLR in adult patients with AS.

## Literature search and data extraction

**Fig. 1:**
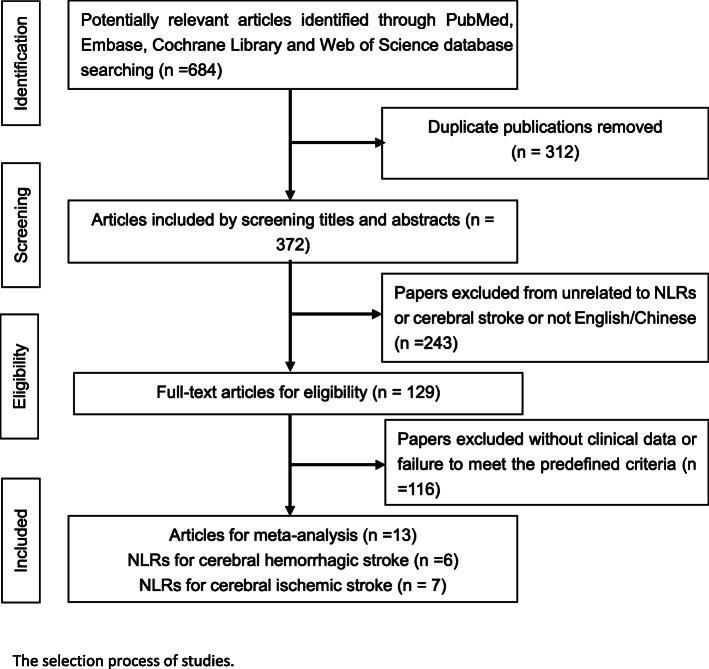
The flowchart of the searching strategy and study selection in the meta-analysis

We carried out a systematic literature search in the PubMed, Web of Science, Cochrane Library, Embase, and China National Knowledge Infrastructure databases (search period: from the establishment of the database to April 16, 2019). The English keywords were as follows: “#1: NLR; #2: Neutrophil–lymphocyte Ratio; #3:Stroke[MeSH]; #4 :Ischemic[MeSH]; #5: Haemorrhagic[MeSH]; #6:[#1or#2]and[#3or#4or#5].” The Chinese keywords were as follows: “#1: NLR; #2: Neutrophil–lymphocyte Ratio; #3:Stroke; #4[(#1or#2)and#3].” The following inclusion criteria were used: (1) the diagnoses were clinically confirmed as cerebral hemorrhage or cerebral ischemic stroke; (2) patients were admitted to the hospital after the onset of the disease and were discharged smoothly (discharged smoothly means that the patient is still alive at the time of discharge). The exclusion criteria included the following: (1) age less than 18; (2) no modified Rankin Scale (mRS) or Glasgow Outcome Scale (GOS) scores at 3 months; (3) lack of relevant data, such as odds ratios (ORs), neutrophil–lymphocyte ratio at admission, or functional outcome at 3 months; (4) routine blood examination was not collected within 24 h after admission; and (5) stroke of a brain tumor. Two reviewers individually extracted the data from these included studies. If there was disagreement about the results of a study, the results would be judged by another reviewer in a discussion. A total of 684 eligible studies were retrieved (up to April 2019). Among them, 312 studies were confirmed to be duplicates and excluded. Then, following a prudent search of the title, abstract, and full text, 243 studies were excluded due to NLR values not reported, not cerebral stroke, no clinical data, or not in English/Chinese. Finally, we included 13 studies in this analysis. The study selection flowchart is shown in Fig. 1.

### Risk of bias assessment

Two researchers assessed bias risk. Publication bias was evaluated by a funnel plot and a significant Egger’s linear regression test at *P* < 0.05.

### Statistical analysis

We used the Stata software version 13.0 (STATA Corporation, USA) for all analyses, using the ORs and the corresponding 95% confidence intervals to estimate the correlation between NLR values and prognosis of adult patients with AS. We preferred to use the adjusted ORs. When the adjusted ORs were absent, we extracted the unadjusted ORs. The quality assessment of all articles included in our study was based on the Newcastle Ottawa scale (NOS) [18]. According to the NOS (0–9 point system), a study with more than 6 points is considered a high-quality study. We used Cochran’s *Q* test and Higgins I-squared statistics (*p* < 0.1 and *I*^2^ > 50%) to test for heterogeneity across the pooled ORs. If *I*^2^ was more than 50%, it was preferable to use a random effects model; if not, the fixed effects model was used instead [19]. Publication bias was determined using filled funnel plots. All of the above data were analyzed by the comprehensive meta-analysis software (Stata 13 MP) with statistical significance indicated at *p* < 0.05.

## Search results and characteristics of the patients

A total of 13 eligible studies with 4443 patients were retrieved (up to April 2019). We make prudent judgements on the eligible data and double-checked the existing research based on the inclusion and exclusion criteria. For each study, the following information was carefully extracted: the first author, country, number of patients (male/female), mean age (years), sampling time of the blood, NLR threshold, outcome measure, adjusted odds ratio (OR), and subtype (Table 1). Among the selected studies, there were 6 hemorrhagic stroke studies [13, 14, 20–23] and 7 ischemia stroke studies [11, 12, 24–28]. The characteristics of the 13 available studies are listed in Table 1. Among the included studies, there were 7 Chinese studies and 6 European and American studies (France, Italy, Greece, Turkey, Germany, and America). Among the 13 studies, 12 studies had poor functional outcome (mRS > 2) at 3 months as the outcome measure [11–14, 17, 20–22, 24, 25, 27, 28], and 7 studies had the outcome measure of death at 3 months [17, 20, 24, 25, 27–29].

**Table 1:** The main characteristics of studies for the prognostic role of the NLR in stroke meta-analysis

Characteristics of the included studies								
Author years	Country	*N* (F/M)	Age, year	Sample time	Optimal cutoff value	Outcome measure	etiology of stroke	treatment modality
Maestrini et al. 2015	France	846 (430/416)	Mean 71	onset< 4.5 h	4.8	mRS at 3 mo	CI	IT
Qun et al. 2017	China	143 (80/63)	Mean 70	admission	2.995	mRS at 3 mo	CI	AP
Xue et al. 2017	China	289 (107/173)	Mean 61.8	admission	2.39	mRS at 3 mo	CI	AG
Duan et al. 2018	China	616 (368/248)	Mean 66	admission < 24 h	7	mRS at 3 mo	LVOS	ET and IT
Goyal et al. 2018	greece	293 (147/146)	Mean 62	admission	NR	mRS at 3 mo	LVOS	MT
Kocaturk et al. 2018	Turkey	107 (57/50)	Mean 67	admission	4.7	death at 3 mo	CI	IT or AP or MT
Malhotra et al. 2018	USA	657 (333/324)	Mean 64.3	admission	2.2	mRS at 3 mo	CI	IT
Lattanzi et al. 2016	Italy	177 (63/114)	Mean 67.1	admission	4.58	mRS at 3 mo	ICH	NR
Tao et al. 2017	China	336 (216/120)	Mean 58.5	admission	4.58 to 7.3 (report)	mRS at 3 mo	sICH	SE
Zhang et al. 2018	China	104 (80/24)	Mean 50.4	admission < 24 h	6.46	mRS at 3 mo	ICH	SE
Sun et al. 2017	China	352 (234/118)	Mean 64.2	admission < 24 h	NR	mRS at 3 mo	ICH	WSE
Qin et al. 2019	China	213 (155/58)	Mean 50	admission < 24 h	NR	mRS at 3 mo	sICH	AG
Giede-Jeppe et al. 2019	Germany	319 (98/221)	Mean 51	admission	7.05	mRS at 3 mo	aSAH	NR

*NR* not reported, *ICH* intracerebral hemorrhage, *CI* cerebral ischemia, *mo* month

### Correlations between NLR levels and prognosis in adult patients with acute stroke

**Fig. 2:**
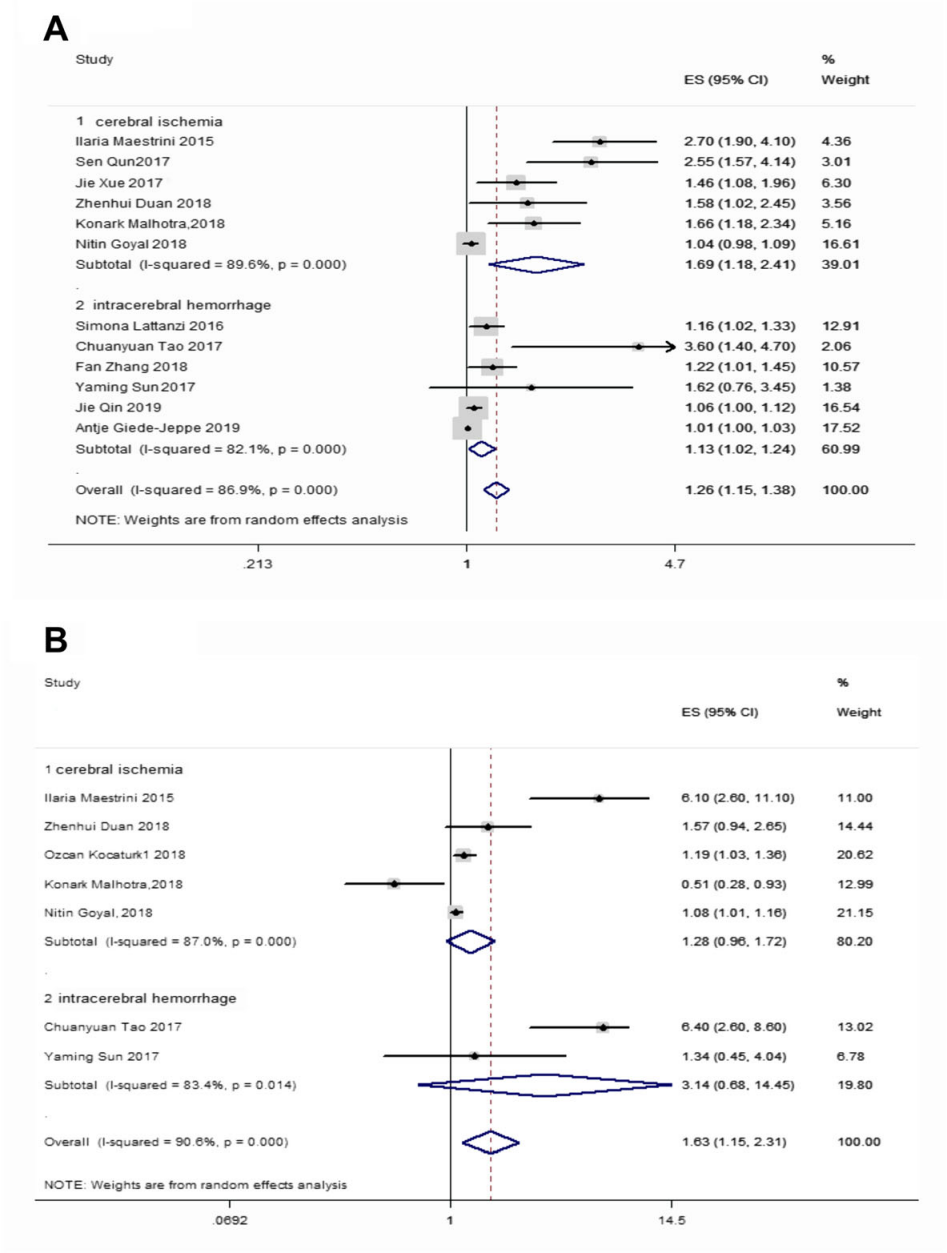
**a** Forest plot of the effects of NLR in the 3-month functional prognosis. **b** Forest plot of the prognostic effect of NLR on a death outcome at 3 months

The pooled ORs of patients for poor outcomes at 3 months were higher in acute ischemic and hemorrhagic patients relative to controls, with values of 1.689 (95% CI = 1.184–2.409, *p* < 0.001) and 1.125 (95% CI = 1.022–1.239, *p* < 0.001), respectively, and the overall pooled OR for acute stroke was 1.257 (95% CI = 1.146–1.379, *p* < 0.001) (Fig. 2a). Meanwhile, the pooled ORs for AS mortality in acute ischemic and hemorrhagic patients at 3 months were 3.142 (95% CI, 0.683–14.455, *p* < 0.001) and 1.282 (95% CI, 0.955–1.720, *p* < 0.014), respectively, and the overall pooled OR following acute stroke was 1.632 (95% CI, 1.155–2.306, *p* < 0.001) (Fig. 2b). This suggests that at 3 months, poor functional outcome in patients with AS is associated with a higher NLR. However, the 3-month mortality in patients with ischemic or hemorrhagic stroke has an uncertain relationship with the NLR. Regarding the results of the 3-month death outcome, neither the ischemic stroke nor the hemorrhagic stroke subgroup analysis was meaningful; however, if the overall effect value following stroke is meaningful, it is not a desirable outcome. All the above details are shown in Table 2.

**Table 2:** Subgroup and meta-regression analysis of ORs on prognostic role of NLR in stroke patients, 95% conf. intervals, and I-squared values

Subgroup analysis	No. of studies	No. of patients	95% conf. interval		Meta-regression (*p* value)	Heterogeneity (random)	
			fixed	random		(*p* value)	***I***^**2**^(%)
poor functional							
Overall pooled OR	12	4336	1.023(1.011–1.036)	1.257 (1.146–1.379)	< 0.001	< 0.001	86.90%
Hemorrhage	6	1501	1.096(1.041–1.152)	1.125(1.022–1.239)	< 0.01	< 0.001	82.10%
cerebral ischemia	6	2835	1.019(1.006–1.032)	1.689(1.184–2.409)	< 0.005	< 0.001	89.60%
mortality							
Overall pooled OR	7	3207	1.131(1.065–1.202)	1.632 (1.155–2.306)	0.207	< 0.001	90.60%
Hemorrhage	2	688	1.11(1.045–1.180)	1.282 (0.955–1.720)	–	< 0.014	87.00%
cerebral ischemia	5	2519	4.473(2.646–7.564)	3.142(0.683–14.455)	0.491	< 0.001	83.40%

### Heterogeneity

**Fig. 3:**
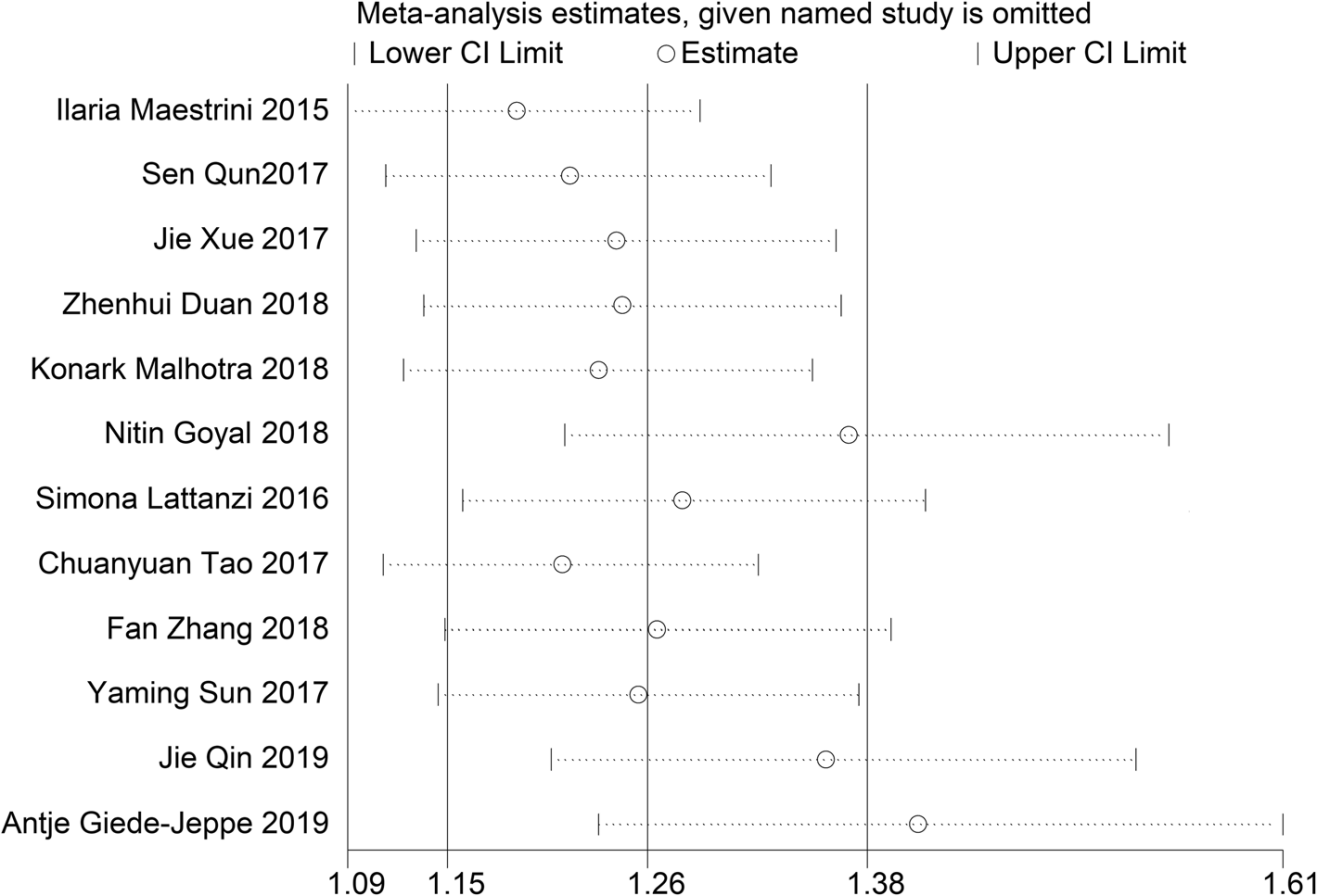
Sensitivity analysis of the effects of NLR at a 3-month functional prognosis

Statistical results showed that there was significant heterogeneity among the studies (*I*^2^ = 86.9%, *p* < 0.0001). We performed a sensitivity analysis (Fig. 3) and found that the studies by Giede-Jeppe et al. [22] and Goyal et al. [25] were significant origins of heterogeneity. After excluding these studies, the heterogeneity dropped by 54.4% (ischemia), 79.2% (hemorrhage), and 85.3% (overall). However, the ORs were still statistically significant (OR 1.877, 95% CI, 1.459–2.415; OR 1.258, 95% CI, 1.047–1.511; and OR 1.574; 95% CI, 1.298–1.910, respectively).

### Publication bias

**Fig. 4:**
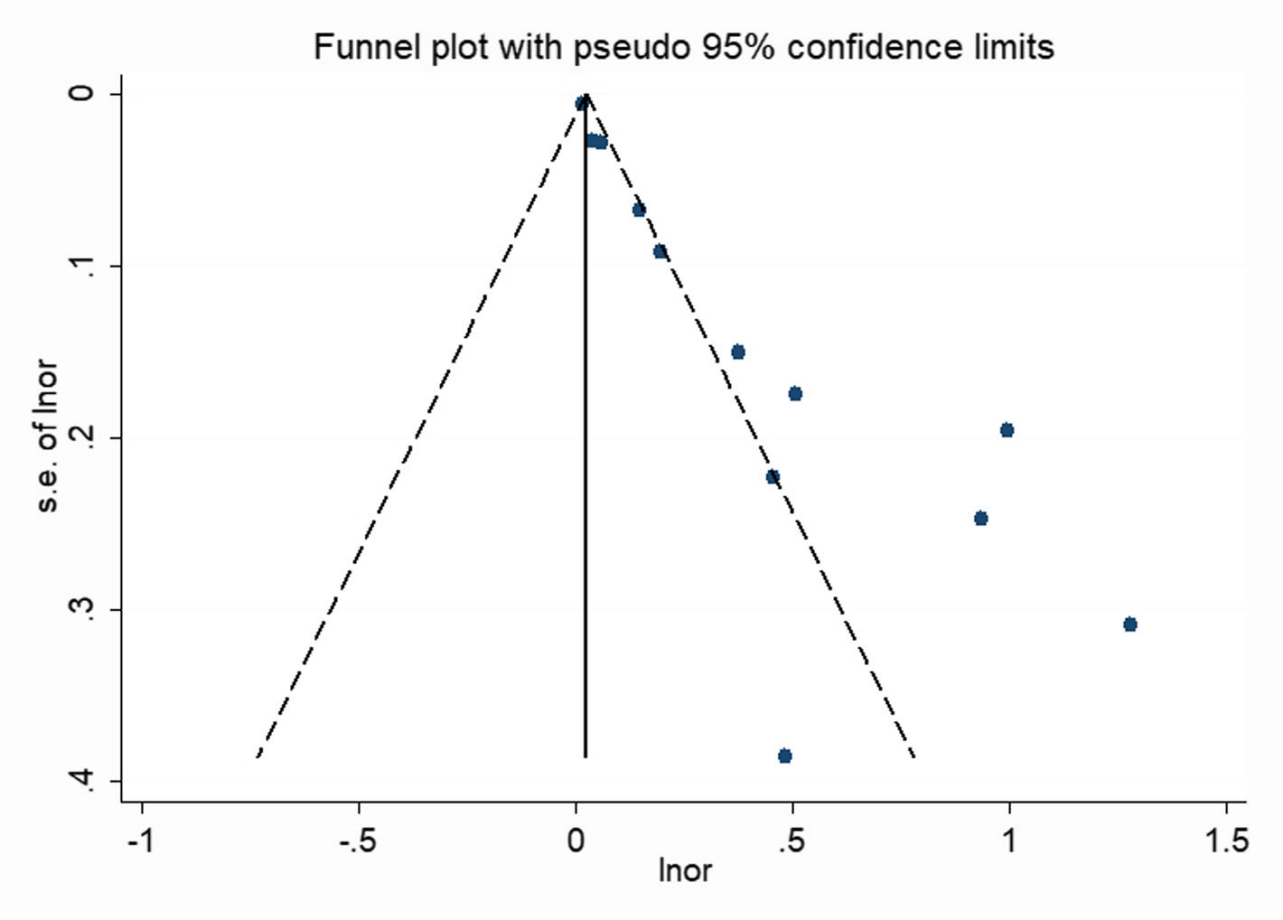
Funnel plot of the effect of NLR on the 3-month functional prognosis

**Fig. 5:**
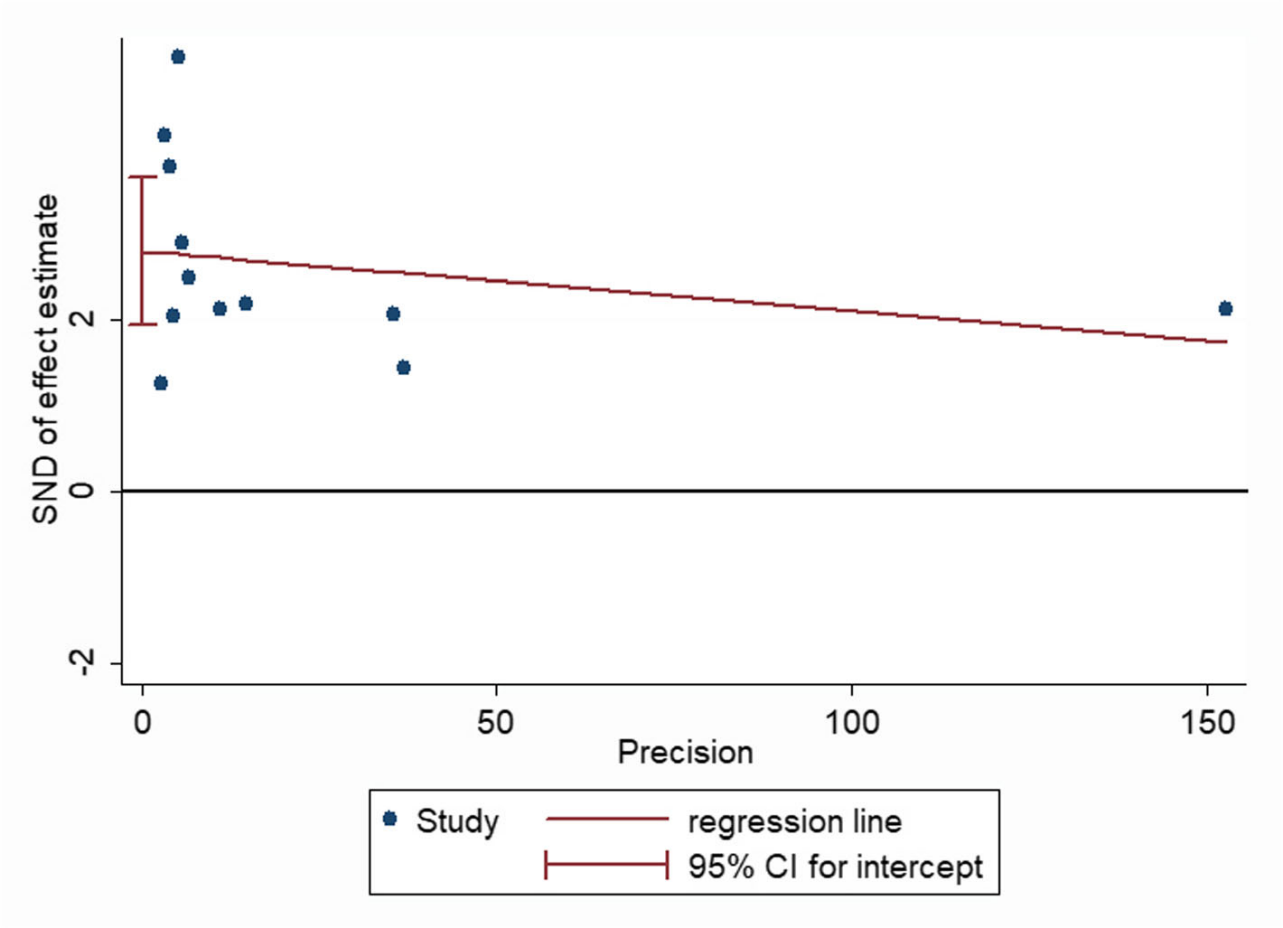
Egger’s funnel plot of the effect of NLR on the 3-month functional prognosis

A significant bias was found in the meta-analysis when we performed a funnel plot of these studies (Fig. 4) and found that the funnel diagram was obviously asymmetric. Therefore, we attempted to determine the reason why there was bias through performing a sensitivity analysis (Fig. 3), Egger’s funnel plot (*p* < 0.001 Fig. 5), and covariate analysis (Supplement Table 1). Egger’s funnel plot showed that the number of studies on the two sides was asymmetric, and the confidence interval did not cross zero. This again indicated the existence of publication bias. A sensitivity analysis showed that the results of Giede Jeppe et al. and Goyal et al. had the greatest impact on the analysis. The results of multiple regression analysis showed that the stroke type (95% CI = 0.616–1.4, *p* = 0.683), study design (95% CI = 0.43–1.46, *p* = 0.409), and country (95% CI = 0.67–2.16, *p* = 0.489), as well as a combined measure (95% CI = 0.806–5.590, *P* = 0.111), were not sources of bias. Finally, we found that publication bias was the primary source of heterogeneity in the meta-analysis.

The purpose of this study was to evaluate the prognostic effect of the NLR in patients 3 months after stroke. We conducted a meta-analysis based on 13 studies to summarize the existing evidence. To the best of our knowledge, this is the first meta-analysis to comprehensively evaluate this question. In our study, 3 months after stroke, a higher NLR was associated with poor functional outcomes, while the relationships between a high NLR and a higher risk of death at 3 months were inconclusive.

We performed subgroup analyses to evaluate the value of the NLR in predicting the functional outcome at 3 months under different conditions. In the poor functional outcome group, the pooled OR of the six studies of ischemic stroke and the pooled OR of the six studies of hemorrhage stroke were both statistically significant, which indicated the applicability of the NLR in these subgroups. With regard to the death outcome, neither the overall OR from the five studies of ischemic stroke nor the OR from the two studies of hemorrhage stroke was statistically significant, indicating that a high NLR may not be related to the mortality of stroke patients at 3 months. Due to the small number of studies and significant heterogeneity, we may need more investigations to further determine the role of a high NLR in predicting the mortality of stroke patients at 3 months.

The inflammatory response plays a dramatic role in stroke. High white blood counts have been related to worse outcome in stroke patients [30]. It has been reported that an increased neutrophil count was associated with more severe stroke on admission [14, 20, 31]. After a stroke, neutrophils rapidly gather around the lesion [32, 33]. Studies have demonstrated that neutrophils can gather at the site of the lesion and cause secondary brain injury [34, 35]. Some researchers have demonstrated that a higher neutrophil count may be due to the release of matrix metalloproteinase-9 by neutrophils [27]. Apoptosis and functional inactivation of lymphocytes is induced by acute central nervous system injury [36], and some studies have shown that lymphocytes can repair inflammation-induced damage [37], which is a leading cause of poor outcome in patients with stroke [31, 36].

The NLR reflects the balance in the relationship between neutrophils and lymphocytes [27, 38], and its assessment could provide a service in clinical work. Of course, there were many other factors associated with the prognosis of stroke patients, such as blood pressure and some biochemical parameters. Neutrophils and lymphocytes are known to reflect inflammatory responses and factors that can be controlled, and therefore, may represent potential therapeutic targets. All the included studies suggested that a higher NLR value had a negative impact on patient outcomes. In our meta-analysis, a higher NLR was associated with poor functional outcome at 3 months, but the relationship with death at 3 months remains unclear. More work will be needed to determine the underlying molecular mechanisms. We failed to prove the correlation between a higher NLR and a high risk of death at 3 months; thus, more studies are needed to clarify this relationship. The NLR is a readily available and inexpensive test that could serve as a predictor of outcome in stroke patients.

We carried out this meta-analysis to demonstrate the implications of the NLR for prognosis in patients with stroke. Our statistical results support the conclusion that the NLR is significantly correlated with poor prognosis in stroke patients. In our results, the pooled ORs of higher NLR for poor functional outcome in acute ischemic and hemorrhagic patients were 1.689 (95% CI = 1.184–2.409, *p* < 0.001) and 1.125 (95% CI = 1.022–1.239, *p* < 0.001), respectively, and the overall pooled OR after acute stroke was 1.257 (95% CI = 1.146–1.379, *p* < 0.001). Moreover, significant D (pooled OR: *I*^2^ = 86.9%, *p* < 0.001; ischemia OR: *I*^2^ = 89.60%, *p* < 0.001; hemorrhage OR: *I*^2^ = 82.10%, *p* < 0.001) due to few studies.

Heterogeneity was found in our meta-analysis of poor functional outcomes. This heterogeneity was probably partially created by geographic area (divided by China or not China), statistical methods, sample size, study design, and stroke type. To determine the source of heterogeneity in this meta-analysis, we used the methods described above. The subgroup analysis results and covariate analysis (Table supplement 1) demonstrated that the predictive value of NLR was not dependent on the factors listed above. Moreover, in the sensitivity analysis (Fig. 3), after omitting Giede-Jeppe et al. [22] or Goyal et al. [25], our results were relatively stable according to the funnel plot and Egger’s test (Fig. 5). The etiology of stroke and treatment modality of Giede-Jeppe et al. and Goyal et al. are part of the source of heterogeneity. The etiology of stroke and treatment modality in other articles in the study are not completely consistent, and some articles do not report treatment methods, which the heterogeneity is also non-negligible. Of course, this needs further study.

Although all blood samples were obtained within 24 h of admission, 7 studies took blood samples within 24 h of stroke onset, and 3 of the studies were within 1 week of stroke onset. Three studies did not mention this, which may have been the source of heterogeneity between the studies. Because the inclusion criteria across studies were different, 5 articles, including Maestrini et al., Malhotra et al., Goyal et al., Lattanzi et al., and Sun et al., did not involve the effects of leukaemia, inflammation, immunosuppression, etc., which may have overestimated the prognostic value of the NLR for stroke patients. In the course of stroke, after the death of brain cells, inflammatory cells will accumulate in damaged parts. The initial inflammatory cells will secrete cytokines, which in turn promote the inflammatory response. This cycle causes cytokine storm, causing brain cell secondary damage. Lymphocytes increased after a few days. At this time, the ratio of neutrophil lymphocytes can well reflect the trend of inflammation after stroke and eliminate the heterogeneity caused by the number of immune cells in some individuals. Therefore, the strong inflammatory response in the early stage after stroke will have a negative impact on the prognosis of stroke.

We rated all articles according to the NOS standard, and the result was that all the included articles were high quality (NOS > 6 points). On the one hand, some conference papers did not provide data; therefore, there were fewer included documents, and positive results were more likely to be published. These are the main reasons for publication bias, so we need more well-designed research to further clarify this issue. Overall, this suggested that publication bias was the primary source of heterogeneity.

**Fig. 6:**
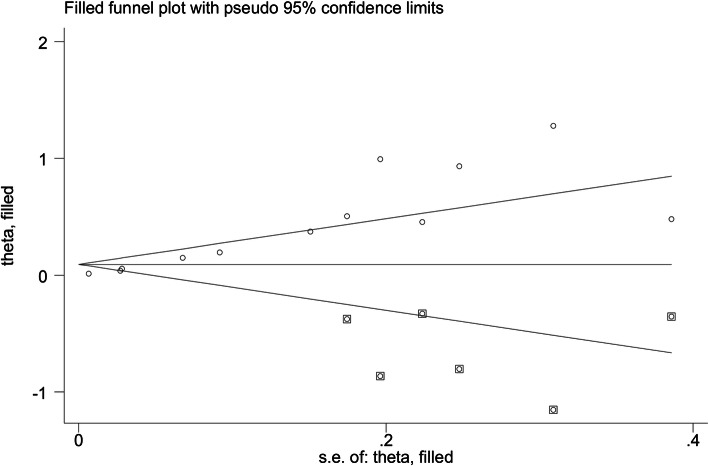
Filled funnel plot for detecting publication bias

Because publication bias was the main source of heterogeneity, the filled funnel plot (Fig. 6) was performed in this study. The filled pooled OR was 0.093 (95% CI, 0.006–0.191, *p* = 0.064), and the original pooled OR was 1.257 (95% CI, 1.146–1.379, *p* < 0.001); the outcome was reversed. Therefore, the result is not very stable. Finally, due to the limitations in the details of each research design, these studies included in the meta-analysis may have the risk of uncertainty bias.

## Limitations

There were several limitations to our study. First, the meta-analysis was based on a limited number of studies. Although we included 13 studies, of these studies, seven were ischemic stroke studies, and 6 were hemorrhagic stroke studies, although cerebral hemorrhage studies and cerebral ischemia studies were not statistically heterogeneous sources. Second, because of the lack of adequate clinical data, we were unable to present a subgroup analysis based on baseline characteristics. In addition, most of the included studies were from Asia (and most were from China), and the overall conclusions should be treated with caution.

## Conclusions

This meta-analysis demonstrates that, for patients with stroke, a higher NLR was associated with poor functional outcome at 3 months, but the relationship with death at 3 months cannot be concluded. This ready-made and cheap marker may be a useful tool in clinical work, which may be helpful for predicting poor functional outcome at 3 months.

## Supplementary information

**Additional file 1: Table S1.** Covariate analysis of this study

## Data Availability

All data generated or analyzed during this study are included in this published article.

## Abbreviations

NLR: Neutrophil–lymphocyte ratio
OR: Odds ratio
GOS: Glasgow Outcome Scale
mRS: Modified Rankin Scale
NOS: Newcastle-Ottawa Scale
NR: Not reported
ICH: Intracerebral hemorrhage
sICH: Spontaneous intracerebral hemorrhage
CI: Cerebral ischemia
mo: Month
AS: Acute stroke
LVOS: Large vessel occlusion strokes
IT: Intravenous thrombolysis
AP: Anti-platelet
AG: According guidelines
ET: Endovascular treatment
MT: Mechanical thrombectomy
SE: Surgical evacuation
WSE: Without surgical evacuation
aSAH: Aneurysmal subarachnoid hemorrhage

## References

[1] Zuhaid M, Salman, Chawla JA, Farooq U, Ahmad A, Khan S, Asfandiyar M (2014). Frequency of modifiable risk factors in stroke patients. J Ayub Med Coll Abbottabad.

[2] Collaborators GBDS. Global, regional, and national burden of stroke, 1990-2016: a systematic analysis for the Global Burden of Disease Study 2016. Lancet Neurol. 2019; 10.1016/S1474-4422(19)30034-1.

[3] Allen CL, Bayraktutan U. Oxidative stress and its role in the pathogenesis of ischaemic stroke. Int J Stroke. 2009; 10.1111/j.1747-4949.2009.00387.x. 10.1111/j.1747-4949.2009.00387.x 19930058

[4] Caceres JA, Goldstein JN. Intracranial hemorrhage. Emerg Med Clin North Am. 2012; 10.1016/j.emc.2012.06.003. 10.1016/j.emc.2012.06.003 PMC3443867 22974648

[5] Broderick JP, Brott TG, Duldner JE, Tomsick T, Huster G (1993). Volume of intracerebral hemorrhage. A powerful and easy-to-use predictor of 30-day mortality. Stroke..

[6] Dowlatshahi D, Demchuk AM, Flaherty ML, Ali M, Lyden PL, Smith EE, Collaboration V. Defining hematoma expansion in intracerebral hemorrhage: relationship with patient outcomes. Neurology. 2011; 10.1212/WNL.0b013e3182143317. 10.1212/WNL.0b013e3182143317 PMC3068004 21346218

[7] Lattanzi S, Silvestrini M, Provinciali L. Elevated blood pressure in the acute phase of stroke and the role of angiotensin receptor blockers. Int J Hypertens. 2013; 10.1155/2013/941783. 10.1155/2013/941783 PMC3574652 23431423

[8] Garton ALA, Gupta VP, Christophe BR, Connolly ES Jr. Biomarkers of functional outcome in intracerebral hemorrhage: interplay between clinical metrics, CD163, and ferritin. J Stroke Cerebrovasc Dis. 2017; 10.1016/j.jstrokecerebrovasdis.2017.03.035. 10.1016/j.jstrokecerebrovasdis.2017.03.035 PMC5513722 28392117

[9] Chen YW, Li CH, Yang CD, Liu CH, Chen CH, Sheu JJ, Lin SK, Chen AC, Chen PK, Chen PL, Yeh CH, Chen JR, Hsiao YJ, Lin CH, Hsu SP, Chen TS, Sung SF, Yu SC, Muo CH, Wen CP, Sung FC, Jeng JS, Hsu CY, Taiwan Stroke Registry I. Low cholesterol level associated with severity and outcome of spontaneous intracerebral hemorrhage: results from Taiwan Stroke Registry. PLoS ONE. 2017; 10.1371/journal.pone.0171379. 10.1371/journal.pone.0171379 PMC5396870 28422955

[10] Sporns PB, Schwake M, Schmidt R, Kemmling A, Minnerup J, Schwindt W, Cnyrim C, Zoubi T, Heindel W, Niederstadt T, Hanning U. Computed tomographic blend sign is associated with computed tomographic angiography spot sign and predicts secondary neurological deterioration after intracerebral hemorrhage. Stroke. 2017; 10.1161/STROKEAHA.116.014068. 10.1161/STROKEAHA.116.014068 27879447

[11] Xue J, Huang WS, Chen XL, Li Q, Cai ZY, Yu T, Shao B. Neutrophil-to-lymphocyte ratio is a prognostic marker in acute ischemic stroke. Journal of Stroke & Cerebrovascular Diseases. 2017; 10.1016/j.jstrokecerebrovasdis.2016.11.010. 10.1016/j.jstrokecerebrovasdis.2016.11.010 27955949

[12] Qun S, Tang Y, Sun J, Liu ZX, Wu JC, Zhang J, Guo JD, Xu ZQ, Zhang D, Chen ZX, Hu FY, Xu XS, Ge W. Neutrophil-to-lymphocyte ratio predicts 3-month outcome of acute ischemic stroke. Neurotox Res. 2017; 10.1007/s12640-017-9707-z. 10.1007/s12640-017-9707-z 28181171

[13] Qin J, Li Z, Gong G, Li H, Chen L, Song B, Liu X, Shi C, Yang J, Yang T, Xu Y. Early increased neutrophil-to-lymphocyte ratio is associated with poor 3-month outcomes in spontaneous intracerebral hemorrhage. PLoS One. 2019; 10.1371/journal.pone.0211833. 10.1371/journal.pone.0211833 PMC6366889 30730945

[14] Lattanzi S, Cagnetti C, Provinciali L and Silvestrini M. Neutrophil-to-lymphocyte ratio predicts the outcome of acute intracerebral hemorrhage. stroke. 2016; 10.1161/strokeaha.116.013627. 10.1161/STROKEAHA.116.013627 27165957

[15] Group GBDNDC. Global, regional, and national burden of neurological disorders during 1990-2015: a systematic analysis for the Global Burden of Disease Study 2015. Lancet Neurol. 2017; 10.1016/S1474-4422(17)30299-5. 10.1016/S1474-4422(17)30299-5 PMC5641502 28931491

[16] O'Donnell MJ, Xavier D, Liu L, Zhang H, Chin SL, Rao-Melacini P, Rangarajan S, Islam S, Pais P, McQueen MJ, Mondo C, Damasceno A, Lopez-Jaramillo P, Hankey GJ, Dans AL, Yusoff K, Truelsen T, Diener HC, Sacco RL, Ryglewicz D, Czlonkowska A, Weimar C, Wang X, Yusuf S and investigators I. Risk factors for ischaemic and intracerebral haemorrhagic stroke in 22 countries (the INTERSTROKE study): a case-control study. Lancet. 2010; 10.1016/S0140-6736(10)60834-3. 10.1016/S0140-6736(10)60834-3 20561675

[17] Sun Y, You S, Zhong C, Huang Z, Hu L, Zhang X, Shi J, Cao Y and Liu C-F. Neutrophil to lymphocyte ratio and the hematoma volume and stroke severity in acute intracerebral hemorrhage patients. American Journal of Emergency Medicine. 2016; 10.1016/j.ajem.2016.11.037. 10.1016/j.ajem.2016.11.037 27876538

[18] Stang A. Critical evaluation of the Newcastle-Ottawa scale for the assessment of the quality of nonrandomized studies in meta-analyses. Eur J Epidemiol. 2010; 10.1007/s10654-010-9491-z. 10.1007/s10654-010-9491-z 20652370

[19] Qian C, Yu X, Li J, Chen J, Wang L, Chen G. The efficacy of surgical treatment for the secondary prevention of stroke in symptomatic moyamoya disease: a meta-analysis. Medicine (Baltimore). 2015; 10.1097/MD.0000000000002218. 10.1097/MD.0000000000002218 PMC5008504 26656359

[20] Tao C, Hu X, Wang J, Ma J, Li H, You C. Admission neutrophil count and neutrophil to lymphocyte ratio predict 90-day outcome in intracerebral hemorrhage. Biomark Med. 2017; 10.2217/bmm-2016-0187. 10.2217/bmm-2016-0187 27917647

[21] Zhang F, Tao C, Hu X, Qian J, Li X, You C, Jiang Y, Yang M. Association of neutrophil to lymphocyte ratio on 90-day functional outcome in patients with intracerebral hemorrhage undergoing surgical treatment. World Neurosurg. 2018; 10.1016/j.wneu.2018.08.010. 10.1016/j.wneu.2018.08.010 30103056

[22] Giede-Jeppe A, Reichl J, Sprugel MI, Lucking H, Hoelter P, Eyupoglu IY, Kuramatsu JB, Huttner HB, Gerner ST. Neutrophil-to-lymphocyte ratio as an independent predictor for unfavorable functional outcome in aneurysmal subarachnoid hemorrhage. J Neurosurg. 2019; 10.3171/2018.9.JNS181975. 10.3171/2018.9.JNS181975 30717052

[23] Sun Y, You S, Zhong C, Huang Z, Hu L, Zhang X, Shi J, Cao Y, Liu CF (2017). Neutrophil to lymphocyte ratio and the hematoma volume and stroke severity in acute intracerebral hemorrhage patients. American Journal of Emergency Medicine..

[24] Duan Z, Wang H, Wang Z, Hao Y, Zi W, Yang D, Zhou Z, Liu W, Lin M, Shi Z, Lv P, Wan Y, Xu G, Xiong Y, Zhu W and Liu X. Neutrophil-lymphocyte ratio predicts functional and safety outcomes after endovascular treatment for acute ischemic stroke. Cerebrovasc Dis. 2018; 10.1159/000489401. 10.1159/000489401 29763889

[25] Goyal N, Tsivgoulis G, Chang JJ, Malhotra K, Pandhi A, Ishfaq MF, Alsbrook D, Arthur AS, Elijovich L, Alexandrov AV. Admission neutrophil-to-lymphocyte ratio as a prognostic biomarker of outcomes in large vessel occlusion strokes. Stroke. 2018; 10.1161/strokeaha.118.021477. 10.1161/STROKEAHA.118.021477 30002151

[26] Kocaturk O, Besli F, Gungoren F, Kocaturk M, Tanriverdi Z (2019). The relationship among neutrophil to lymphocyte ratio, stroke territory, and 3-month mortality in patients with acute ischemic stroke. Neurological Sciences..

[27] Maestrini I, Strbian D, Gautier S, Haapaniemi E, Moulin S, Sairanen T, Dequatre-Ponchelle N, Sibolt G, Cordonnier C, Melkas S, Leys D, Tatlisumak T, Bordet R. Higher neutrophil counts before thrombolysis for cerebral ischemia predict worse outcomes. Neurology. 2015; 10.1212/WNL.0000000000002029. 10.1212/WNL.0000000000002029 PMC4626239 26362283

[28] Malhotra K, Goyal N, Chang JJ, Broce M, Pandhi A, Kerro A, Shahripour RB, Alexandrov AV, Tsivgoulis G. Differential leukocyte counts on admission predict outcomes in patients with acute ischaemic stroke treated with intravenous thrombolysis. Eur J Neurol. 2018; 10.1111/ene.13741. 10.1111/ene.13741 29953701

[29] Kocaturk O, Besli F, Gungoren F, Kocaturk M, Tanriverdi Z. The relationship among neutrophil to lymphocyte ratio, stroke territory, and 3-month mortality in patients with acute ischemic stroke. Neurol Sci. 2018; 10.1007/s10072-018-3604-y. 10.1007/s10072-018-3604-y 30327959

[30] Furlan JC, Vergouwen MD, Fang J, Silver FL. White blood cell count is an independent predictor of outcomes after acute ischaemic stroke. Eur J Neurol. 2014; 10.1111/ene.12233. 10.1111/ene.12233 23848934

[31] Kim J, Song TJ, Park JH, Lee HS, Nam CM, Nam HS, Kim YD, Heo JH. Different prognostic value of white blood cell subtypes in patients with acute cerebral infarction. Atherosclerosis. 2012; 10.1016/j.atherosclerosis.2012.02.042. 10.1016/j.atherosclerosis.2012.02.042 22460048

[32] Kleinig TJ, Vink R (2009). Suppression of inflammation in ischemic and hemorrhagic stroke: therapeutic options. Curr Opin Neurol..

[33] Aronowski J, Zhao X. Molecular pathophysiology of cerebral hemorrhage: secondary brain injury. Stroke. 2011; 10.1161/STROKEAHA.110.596718. 10.1161/STROKEAHA.110.596718 PMC3123894 21527759

[34] Hartl R, Schurer L, Schmid-Schonbein GW, del Zoppo GJ. Experimental antileukocyte interventions in cerebral ischemia. J Cereb Blood Flow Metab. 1996; 10.1097/00004647-199611000-00004. 10.1097/00004647-199611000-00004 8898682

[35] Sansing LH, Harris TH, Kasner SE, Hunter CA, Kariko K. Neutrophil depletion diminishes monocyte infiltration and improves functional outcome after experimental intracerebral hemorrhage. Acta Neurochir Suppl. 2011; 10.1007/978-3-7091-0693-8_29. 10.1007/978-3-7091-0693-8_29 PMC3702167 21725751

[36] Meisel C, Schwab JM, Prass K, Meisel A, Dirnagl U. Central nervous system injury-induced immune deficiency syndrome. Nat Rev Neurosci. 2005; 10.1038/nrn1765. 10.1038/nrn1765 16163382

[37] Schwartz M, Moalem G. Beneficial immune activity after CNS injury: prospects for vaccination. J Neuroimmunol. 2001; 10.1016/s0165-5728(00)00447-1. 10.1016/s0165-5728(00)00447-1 11164901

[38] Zhu W, Guo Z, Yu S. Higher neutrophil counts before thrombolysis for cerebral ischemia predict worse outcomes. Neurology. 2016; 10.1212/01.wnl.0000481976.41273.a1. 10.1212/01.wnl.0000481976.41273.a1 26976518

