# Supplementary material for: The predictive role of the neutrophil–lymphocyte ratio in the prognosis of adult patients with stroke
Source: Chin Neurosurg J. 2020 Jul 1;6:22. doi: 10.1186/s41016-020-00201-5 (PMC7398197; doi:10.1186/s41016-020-00201-5)
Supplement: Supplementary file 1 — Additional file 1: Table S1. Covariate analysis of this study [file 41016_2020_201_MOESM1_ESM.docx]

| Covariate analysis | | | |
| --- | --- | --- | --- |
| Type | P value | Lci | Uci |
| design type | 0.683 | 0.616 | 1.4 |
| stroke type | 0.409 | 0.43 | 1.46 |
| country | 0.489 | 0.67 | 2.16 |
| Lci: low confidence interval; Uci: up confidence interval; country: China or not China | | | |
